# Research Trends around Exercise Rehabilitation among Cancer Patients: A Bibliometrics and Visualized Knowledge Graph Analysis

**DOI:** 10.1155/2022/3755460

**Published:** 2022-05-27

**Authors:** Yun Pan, Xianyu Deng, Ying Zhuang, Jiyu Li

**Affiliations:** ^1^Department of General Surgery, Tenth People's Hospital of Tongji University, Tongji University School of Medicine, Shanghai 200072, China; ^2^Department of Neurosurgery, Tenth People's Hospital of Tongji University, Tongji University School of Medicine, Shanghai 200072, China; ^3^Colorectal Cancer Center, Shanghai Tenth People's Hospital, Shanghai 200072, China; ^4^Geriatric Cancer Center, Huadong Hospital Affiliated to Fudan University, Shanghai 200040, China

## Abstract

This study analyzed the research hotspots and frontiers of exercise rehabilitation among cancer patients via CiteSpace. Relevant literature published in the core collection of the Web of Science (WoS) database from January 1, 2000, to February 6, 2022, was searched. Further, we used CiteSpace5.8R1 to generate a network map and identified top authors, institutions, countries, keywords, and research trends. A total of 2706 related literature were retrieved. The most prolific writer was found to be Kathryn H Schmitz (21 articles). The University of Toronto (64 articles) was found to be the leading institution, with the United States being the leading country. Further, “rehabilitation,” “exercise,” “quality of life,” “cancer,” and “physical activity” were the top 5 keywords based on frequency; next, “disability,” “survival,” “fatigue,” “cancer,” and “rehabilitation” were the top 5 keywords based on centrality. The keyword “fatigue” was ranked at the top of the most cited list. Finally, “rehabilitation medicine,” “activities of daily living,” “lung neoplasm,” “implementation,” “hospice,” “exercise oncology,” “mental health,” “telemedicine,” and “multidisciplinary” are potential topics for future research. Our results show that the research hotspots have changed from “quality of life,” “survival,” “rehabilitation,” “exercise,” “cancer,” “physical therapy,” “fatigue,” and “breast cancer” to “exercise oncology,” “COVID-19,” “rehabilitation medicine,” “inpatient rehabilitation,” “implementation,” “telemedicine,” “lung neoplasm,” “telehealth,” “multidisciplinary,” “psycho-oncology,” “hospice,” “adapted physical activity,” “cancer-related symptom,” “cognitive function,” and “behavior maintenance.” Future research should explore the recommended dosage and intensity of exercise in cancer patients. Further, following promotion of the concept of multidisciplinary cooperation and the rapid development of Internet medical care, a large amount of patient data has been accumulated; thus, how to effectively use this data to generate results of high clinical value is a question for future researchers.

## 1. Introduction

According to data released by the International Agency for Research on Cancer (IARC) in December 2020, there were 19.29 million new cancer cases and 9.96 million cancer-related deaths worldwide in 2020, with this then bringing about heavy health and social burdens. Furthermore, the number of new cancer cases and related deaths in China ranked first globally [1]. Therefore, the associated health problems and social concerns caused by cancer are particularly burdensome for this country.

Effective cancer diagnoses can be advanced with improved medical conditions, which are reflected in the fact that disease-related mortality and progression-free survival cannot be used to reflect multidimensional treatment effects and patient survival statuses fully. Herein, patients have high requirements and expectations in terms of both their treatment and rehabilitation. As such, quality of life (QOL) is increasingly being used as a primary outcome measure for evaluating cancer treatment effects, long-term survival, and functional status among survivors in contemporary research [2, 3].

Exercise is one method that has been found to improve the prognosis of numerous cancer patients and increase their overall QOL [3–5]. The World Health Organization (WHO), the Centers for Disease Control and Prevention (CDC), the American Cancer Society (ACS), and the National Cancer Institute (NCI) all recommend that cancer survivors maintain adequate levels of physical activity after treatment to maintain their overall health [6–9]. Previous studies have also outlined the critical role of exercise as a “prescription” for various chronic noncommunicable diseases [10–12].

Due to an extraordinary abundance of literature on this topic, the classification and summary of related research using more traditional reading methods often have certain limitations. Following the improvement of computer technologies and bibliometrics, software to visualize document information that allows for the drawing and analysis of scientific data has been gaining increased attention. In this context, bibliometrics [13, 14], visualized scientific knowledge graphs [6, 15, 16], and their related applications in bibliometrics are no longer the exclusive branches of information science and information management science. They are also being applied within the natural science, humanities, and societal concerns. In the context of scientometrics, methods for data and information visualization—as based on Thomas Samuel Kuhn's paradigm [7–9], Derek John de Solla Price's s scientific frontier theory, Bo Ronald S. Burt's structural hole theory [17], Kleinberg's burst detection technology, the optimal foraging theory (OFT) [18–21], and Chinese knowledge unit discrete and reorganization theory [22]—have been produced and developed by CiteSpace [23, 24] and other citation visualization analysis software. With the rapid growth of contemporary scientific research and published literature, visual analysis software provides a strong convenience for scientific researchers to use existing data to refine and extract new knowledge. Kuhn believes that the essence of scientific development is the iterative movement process of conventional science and scientific revolution, as well as between the accumulation paradigm and cross-revolution paradigm; as such, examining both emerging and classic literature provides a turning point for science as a whole. Derek John de Solla Price's scientific frontier theory is based on both Bernard's “Network Thinking of Scientific Development Model” and the Science Citation Index (SCI) and assumes that the research frontier is based on recent research results. Further, structural holes are measured in CiteSpace using the betweenness centrality of nodes in the more comprehensive network; herein, a sudden increase in frequency indicates the criticality of a given research topic. The original knowledge map was based on the laws of scientific development in mathematical equations and was then used to display simple curves or two-dimensional graphics. The best information foraging theory and Hidden Markov Model (HMM) [25] provide a new foundation for the vision-oriented integration strategy. With the help of the knowledge map as optimized by the presentation model, researchers can now see through the complex structures of various fields into the broader knowledge system, allowing them to clarify the complex knowledge networks formed by information explosions. The use of computer software to process documents using bibliometrics, visualized scientific knowledge maps, etc. can either replace or enhance scientific researchers' repetitive mental work and further liberate and stimulate scientific research productivity [26].

To understand the research results and trends of the effects of exercise therapy in cancer rehabilitation, we used bibliometrics and visual knowledge graph analytical methods to display the relevant research status allowing for the exploration of novel development laws and new research hotspots. The core concepts of CiteSpace—the visualization research software used in this study—include burst detection, betweenness centrality, and heterogeneous networks, which all help to visualize ongoing research frontiers, research statuses, hotspots, and the prompt discovery of new research trends [23, 27].

## 2. Materials and Methods

### 2.1. Literature Sources and Search Strategies

#### 2.1.1. Literature Sources

In this study, the core collection of the Web of Science (WoS) database was used as its source of the literature, with the last updated search time being on February 6, 2022. As a global authoritative citation database, WoS includes more than 11,000 authoritative and high-impact international academic journals, covering the fields of the natural sciences, biomedicine, engineering technology, social sciences, arts, humanities, etc. Its powerful analysis function also helps researchers to more effectively grasp relevant topics and search for breakthroughs and innovations in research. Furthermore, it allows them to find high-impact papers quickly, discover research directions that their peers are concerned with, and identify the development of research trends in a given field. These characteristics make WoS a popular database for bibliometric research. WoS also contains references cited in papers and compiles a unique citation index that includes the author, article source, and year of publication [28]. Therefore, this study adopted WoS as its database.

#### 2.1.2. Search Strategies

We conducted our searches according to the retrieval strategies shown in Table 1 and collected articles from the core collection of the WoS database from January 1, 2000, to February 6, 2022. The search terms used include “exercise,” “tumor,” and “recovery.”

### 2.2. Inclusion and Exclusion Criteria

We downloaded and imported the retrieved information into the literature management software EndNote 20, which included primary research, clinical research, and reviews related to exercise-based rehabilitation for cancer patients. We then eliminated duplicate literature, patents, seminar reports, conference abstracts, edited materials, and other nonresearch papers. Studies related to animal experiments were also been excluded.

### 2.3. Visualization and Statistical Analyses

Documents that satisfied our inclusion criteria were included in text format, along with their full documentation and citations that were in turn named “download_1-5.” The output documents were then imported into the visual analysis software CiteSpace5.8.R1. After time slicing and thresholding, modeling, pruning, merging, mapping, and other analytical steps [29], the subject headings, authors, author units, countries or regions of origin, date of publication, and additional information were extracted for analysis. The results are presented in graphs, with different nodes representing various elements, including factors like countries, institutions, and keywords, with their sizes reflecting the number or frequency of publications herein [30]. Links between nodes represent the relationship between them, including factors like collaboration, co-occurrence, or coreference. The colors of the nodes and the connecting lines represent different clusters or years [31]. In addition, the centrality index was used to evaluate each source's journal, author, institution, or country. In network maps, a node with a high degree of centrality indicates that it is highly connected to other nodes or lies between two different sets of nodes. The purple outer ring reflects a node with a centrality greater than 0.1, which indicates that it has a significant impact [32]. The resulting data were analyzed using Microsoft 365 Excel and IBM SPSS Grad Pack v27 [33]. A *p*-value of <0.05 was considered statistically significant.

## 3. Results and Discussion

### 3.1. Literature Analysis

**Figure 1:**
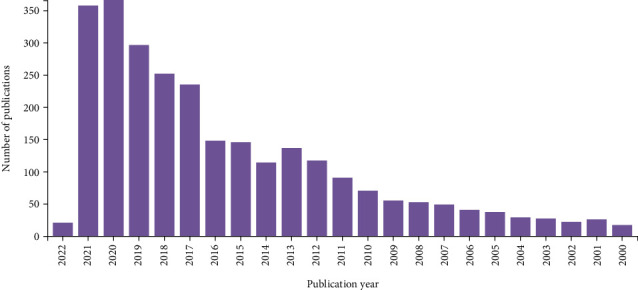
Annual trends in the number of publications from 2000 to 2022.

We included 2706 articles that satisfied our criteria, with the results showing a fluctuating upward trend in the number of publications from 2000 to 2022. From 2016 to 2017, specifically, the number of related publications showed its largest increase (87 articles), indicating that, from 2016, increasing numbers of researchers began to focus on exercise-based rehabilitation in cancer patients. However, when compared with the data in 2020, the number of related publications in 2021 decreased by 8, which was likely due to the COVID-19 pandemic in Europe and the United States, which resulted in a reduced production of research in regions and countries that would have typically produced more articles (Figure 1). In a trend analysis of the number of publications over the study period, we found that the increase in publications was statistically significant overall (*p* = 0.006).

### 3.2. Collaborative Network of Countries, Institutions, and Authors

**Figure 2:**
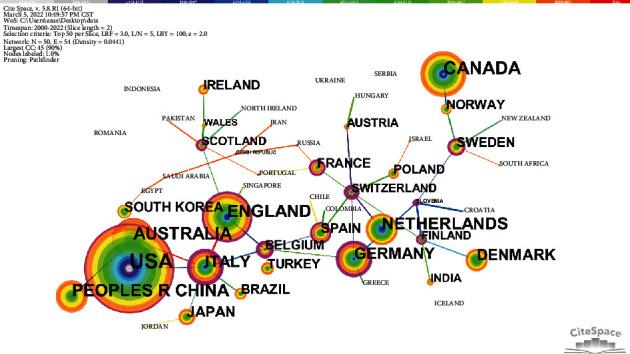
The co-country network of co-country.

As shown in our visualization, the top 5 countries with the most significant number of publications are the United States (729 articles), Canada (273 articles), the United Kingdom (230 articles), Australia (222 articles), and the People's Republic of China (221 articles). Among these, the centrality of the United States and the United Kingdom both exceed 0.1, indicating the essential contributions of these two countries in this field. Conversely, although Canada, Australia, and China are also in leading positions in terms of their number of articles published, the centrality of these countries is low, which indicates that the quality of articles published in these regions still has to be improved (Figure 2).

**Figure 3:**
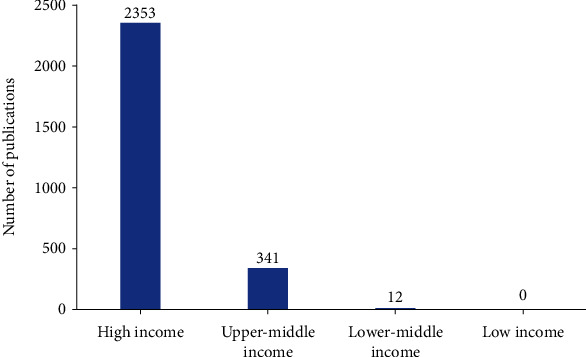
Number of articles published across the different international economic groups.

According to the new regulations for the division of countries based on different income groups as announced by the World Bank on July 1, 2021, nations have been divided into low income (gross national income per capita, GNIPC <1046 US dollars), lower-middle income (GNIPC 1046-4095 US dollars), upper-middle income (GNIPC 4096-12695 US dollars), and high income (GNIPC >12695 US dollars). Among these, high-income countries have contributed more than three-quarters of the total publications (2,353) internationally, which is then followed by upper-middle income countries (341) and lower-middle income countries (12), with no publications on this topic originating from low-income countries (Figure 3) [34]. This phenomenon means that a country's economic situation affects its publishing ability.

**Figure 4:**
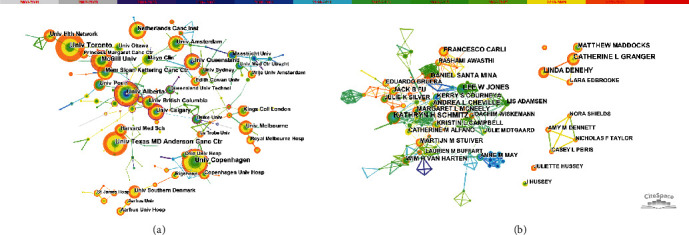
(a) The co-institution network; (b) the co-author network.

Research institutions with the highest number of publications include the University of Toronto, Canada (64 articles), the University of Copenhagen, Denmark (58 articles), the University of Alberta, Canada (58 articles), the University of Texas MD Anderson Cancer Center, USA (55 articles), and McGill University, Canada (50 articles) (Figure 4(a)). The inter-agency cooperation relationship herein shows a relatively localized trend, indicating that multiagency and cross-regional cooperation need to be strengthened.

Figure 4(b) depicts the network of the co-authors. The author with the highest number of papers is Kathryn H Schmitz from the University of Pennsylvania, USA, with 21 articles, who is then followed by Catherine L Granger from the University of Melbourne, Australia (19 articles), and Lee W Jones from Memorial Sloan Kettering Cancer Center, USA (19 articles). Like institutional cooperation, the author cooperation network also shows a localization trend, with the centrality of authors being low overall, indicating that sufficient, extensive, and high-quality collaboration between authors needs to be strengthened in future.

### 3.3. Keyword Co-Occurrence and Cluster Analysis

**Figure 5:**
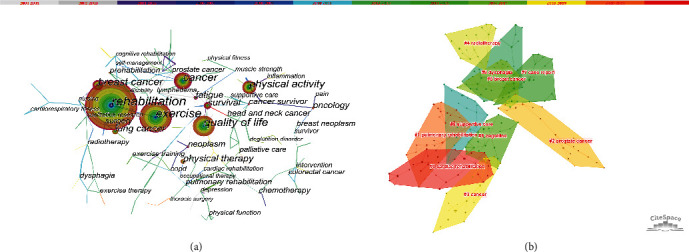
(a) The co-occurring keywords network; (b) the co-occurring keywords cluster network.

Figure 5(a) depicts the co-occurring keywords network; in order to analyze the co-occurrence of keywords more clearly, here Table 2 lists the top 10 keywords in terms of their high frequency and centrality. These keywords reflect ongoing research hotspots within the topic of exercise-based rehabilitation for cancer patients. The co-occurring keywords were analyzed using a log-likelihood ratio to generate 10 clusters, which included the following: “cardiac rehabilitation,” “pulmonary rehabilitation,” “prostate cancer,” “cancer,” “radiotherapy,” “breast cancer,” “cognition,” “case report,” “dysphagia,” and “supportive care” (Figure 5(b)).

### 3.4. The Evolution of Keywords over Time

**Figure 6:**
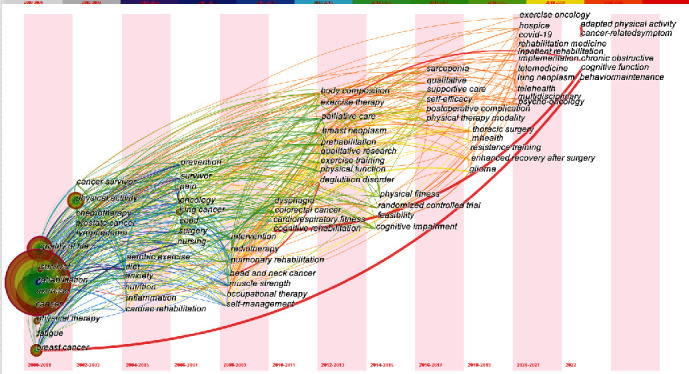
Time zone network for the keywords.

The temporal evolution of keywords in the included literature was also analyzed. On the whole, the hotspots changed from “quality of life,” “survival,” “rehabilitation,” “exercise,” “cancer,” “physical therapy,” “fatigue,” and “breast cancer” to “exercise oncology,” “COVID-19,” “rehabilitation medicine,” “inpatient rehabilitation,” “implementation,” “telemedicine,” “lung neoplasm,” “telehealth,” “multidisciplinary,” “psycho-oncology,” “hospice,” “adapted physical activity,” “cancer-related symptom,” “cognitive function,” and “behavior maintenance.” The research on cancer has expanded from early studies of breast cancer to those on other types of tumors. Due to the rapid development of the Internet, medical treatment has also shifted from being primarily short-distance to a combination of near-telemedicine treatment, with greater emphasis being placed on the concept of precision treatment (Figure 6).

**Figure 7:**
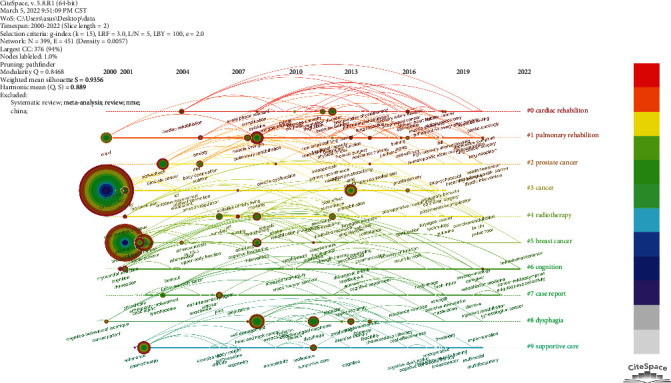
Timeline network of the keywords.

In a timeline plot containing the cluster information (Figure 7), a shift is seen in research hotspots from cardiorespiratory function to generalized physical function and from earlier research on depression and anxiety to an emphasis on supportive care. In addition, recent studies on enhanced recovery after surgery and elderly patients are likely to become a new research hotspot.

**Figure 8:**
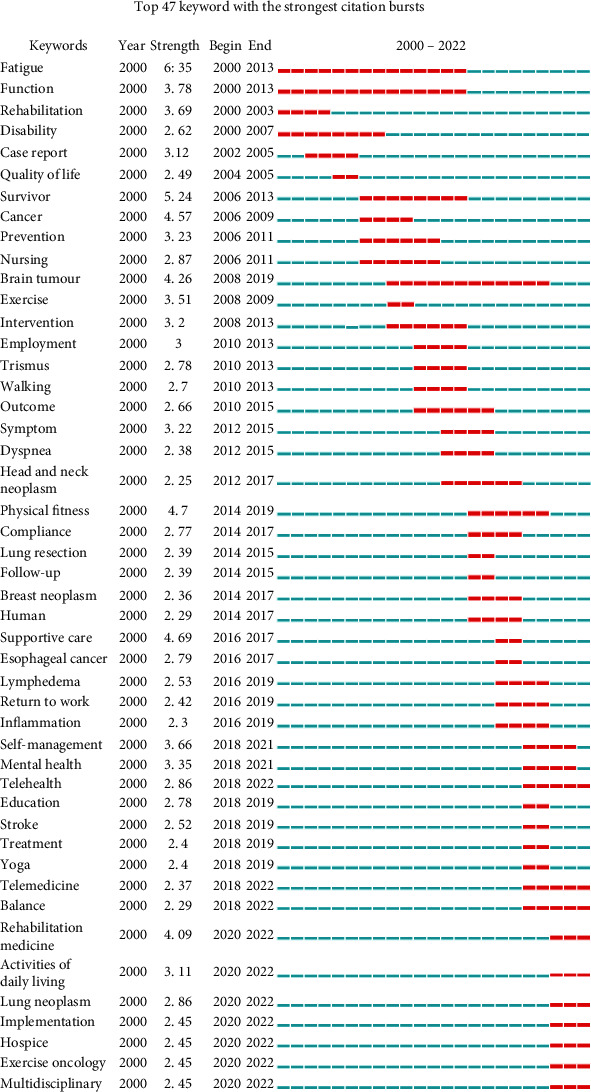
Top 47 keywords with strongest citation bursts.

Figure 8 shows the top 47 keywords with the strongest citation bursts. The segmented blue line represents the time interval, with the red line representing the most substantial period of each keyword's burst [35]. The top three keywords with the strongest citation bursts are “fatigue,” “survivor,” and “physical fitness,” indicating that the symptoms, recovery, and long-term survival of cancer patients have consistently been the focus of research by experts in the field of oncology rehabilitation. The three keywords “fatigue,” “function,” and “disability” arose early in the dataset and lasted for around ten years. Another keyword that appeared in the early stage was “rehabilitation”; however, it only lasted for three years. The reason for this may be that rehabilitation medicine had not been well developed, meaning that research related to it has not been carried out smoothly. The most cited keywords in terms of the bursts appearing in 2020 are “rehabilitation medicine,” “activities of daily living,” “lung neoplasm,” “implementation,” “hospice,” “exercise oncology,” and “multidisciplinary.” All of these then continued into 2022 and may even become new hotspots in the field. In addition, researchers' focus on the keywords of “telehealth,” “telemedicine,” and “balance” has also continued into 2022.

## 4. Conclusions

We used CiteSpace to perform a bibliometric analysis of 2706 global scientific results on exercise rehabilitation for use among cancer patients published from 2000 to 2022 using multiple perspectives, following which we presented the results in knowledge network maps and tables. This study's results show that, from 2000 to 2022, academic attention in exercise rehabilitation for use among cancer patients has experienced a steady growth trend and will likely continue to receive a good deal of attention with the ongoing development of rehabilitation medicine. In addition, it is worth noting that the roles of adapted physical activity, cognitive function, and behavior maintenance in the research body of this field are becoming increasingly prominent, which means that they may become prospective avenues for the development of this field. In addition, the most productive author found was Kathryn H Schmitz from the United States. Additionally, the United States and Canada are the two most significant contributors herein. Correspondingly, most of the institutions with a large number of publications are from these two countries, among which the University of Toronto and the University of Texas MD Anderson Cancer Center are among the top in terms of their number of publications. Our examination into keywords with citation bursts showcased that “rehabilitation medicine,” “activities of daily living,” “lung neoplasm,” “implementation,” “hospice,” “exercise oncology,” “mental health,” “telemedicine,” and “multidisciplinary” may be potential directions for future research.

As early as 1993, Hoffman [36] mentioned the phrase: “exercise is medicine” (EIM) in a paper, with this concept then being widely used in the prevention and treatment of various chronic noncommunicable diseases. In 2007, the American Medical Association (AMA) and the American College of Sports Medicine (ACSM) jointly launched an EIM project focusing on increasing participants' physical activity (PA) and promoting their health through the use of appropriate exercises [37]. The critical role and clinical recommendations of EIM in terms of treating tumors have also attracted increasing attention. The United States is the country with the most significant number of publications herein, which may be related to its earlier proposals and initiatives. In addition, in the latest data released by the World Bank, the United States ranks first in terms of its gross national income per capita and gross domestic product. Further, the literature has confirmed that there is a significant positive correlation between national economic size and scientific productivity [34, 38], which may be another reason why the United States is the country with the largest number of publications in this field. Although the total number of articles published by Chinese scholars ranks in the forefront of this area, the number of articles published by its core authors is relatively small, with the centrality of its cooperation network being low. As such, increased encouragement and promotion of academic exchange activities among scholars in related fields across different countries, as well as the development of domestic and foreign cooperative relations, the improved scientific research, the sharing of research results, and the joint promotion of the development of exercise rehabilitation among cancer patients, are all needed.

**Figure 9:**
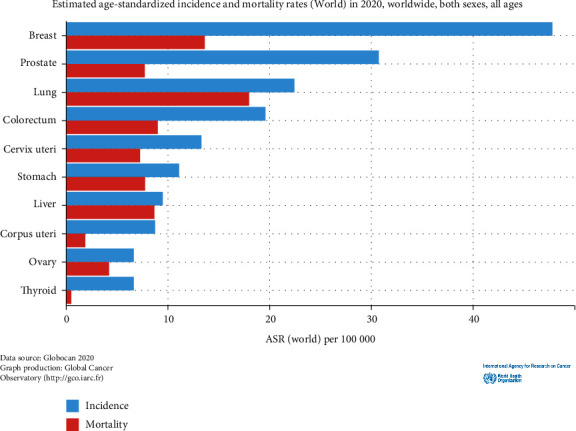
Estimated international age-standardized incidence and mortality rates of various cancer types in 2020 across both sexes and all ages. Source: IARC data visualization exploration [1, 39, 40].

**Figure 10:**
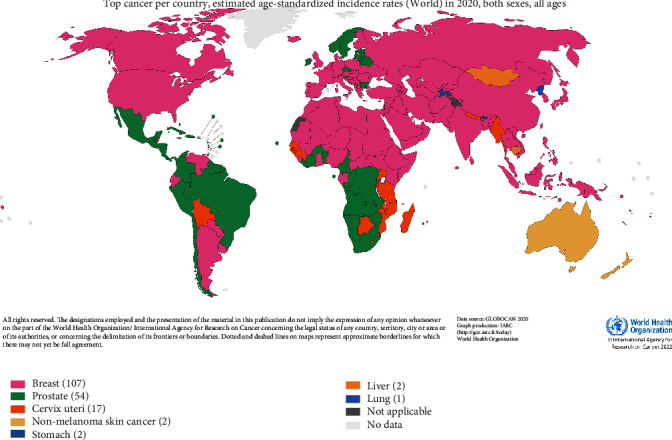
Top cancer type per country and the international estimated age-standardized incidence rates in 2020 for both sexes and all ages. Source: IARC data visualization exploration [1, 39, 40].

From our analysis of keywords, certain cancer types (breast, lung, and prostate cancer) received sufficient attention and research in the early stage of this field's development, which may be related to the high incidence and mortality of these three cancers in the wider population (Figure 9) [1, 39, 40]. In terms of the (estimated age standardized) incidence rates worldwide, these are also the three most common cancer types overall. The results of our bibliometric analysis revealed that, when compared with other types of cancer, there are more research reports related to these three malignant tumors, with the research hotspots about them having developed at an earlier stage. The results of our bibliometric analysis are consistent with the findings of international statistics. Breast and prostate cancer are the “number one cancers” with the highest incidence rates in most countries internationally (Figures 10) [1, 39, 40], which is enough to draw the attention of local medical institutions and health policy departments in their investment in research related to these two cancers.

QOL is another concept that is generally emphasized in the literature. For example, the fatigue-related symptoms of cancer patients have been researched in detail, with there being more studies linking specific cancer types with physical therapy. However, only high-intensity interval training has been studied for use within specific exercise intervention methods. An earlier paper [41] reviewed various types of cancer survivors who exercised regularly, such as through the use of endurance and aerobic training, supervised and home training, recreational PA, and nonrecreational PA, with them finding that PA reduces the risk of certain cancers. Herein, survivors are able to reduce their risk of disease recurrence and increase their chance of prolonged survival, in addition to improving their overall QOL, both during and after treatment. However, cancer survivors still generally lack any significant levels of PA. Furthermore, many medical professionals, patients, and caregivers lack knowledge of exercise programs, doubt the safety or suitability of exercise programs, or have a low compliance with guidelines [42]. As such, more research on different exercise intervention methods is needed to provide more diversified and reliable treatment options.

The types of studies included in our literature search include systematic reviews, qualitative studies, and controlled trials, among which clinical trials were most prominent in 2010-2018. While promoting the concept of EIM in clinical practice after considering its potential benefits for patients, how to further explore and obtain high-quality, evidence-based medical evidence has become a problem to which scientific researchers should pay more attention.

This study does possess several limitations, such as the fact that the core collection of the WoS database was the only data source used due to its inherent advantages, meaning that other databases like PubMed, Scopus, and Google Scholar were not included. Second, there was a lack of non-English database literature and a potential bias resulting from our use of self-citation. Nonetheless, our study comprehensively outlines the current status of research on exercise rehabilitation, as well as the frontiers aimed at cancer patients that have developed from 2000 to 2022, which then provides possible directions for future research. Therefore, the bibliometric analysis of this study will be helpful to relevant scholars, clinicians, and students.

## Figures and Tables

**Table 1:** Search strategies.

Set	Search formula
#1	TS = (exercise∗or^“^physical activity∗^”^ or aerobic∗or walk∗or endurance∗or training or yoga or pilates or tai ji or tai chi or tai − ji or tai − chi or taiji∗)
#2	TS = (cancer∗or tumor∗or tumour∗or neoplas∗or malignan∗or carcinoma∗or adenocarcinoma∗or choricarcinoma∗or leukemia∗or leukaemia∗or metastat∗or sarcoma∗or teratoma∗or melanoma∗or lymphoma∗or myeloma∗or glioma∗or glioblastoma∗or cystadenocarcinoma∗or mesothelioma∗or neuroblastoma∗or osteosarcoma)
#3	TS = (rehabilitate∗)
#4	#2 AND #1
#5	#3 AND #4

Note. TS= Topic Search.

**Table 2:** Top 10 keywords in terms of their frequency and centrality.

Rank	Frequency	Keyword	Centrality	Keyword
1	772	Rehabilitation	0.77	Disability
2	498	Exercise	0.74	Survival
3	351	Quality of life	0.72	Fatigue
4	294	Cancer	0.61	Cancer
5	238	Physical activity	0.6	Rehabilitation
6	195	Breast cancer	0.59	Exercise
7	126	Lung cancer	0.48	Function
8	125	Survival	0.47	Neoplasm
9	123	Physical therapy	0.45	Breast cancer
10	95	Fatigue	0.42	Nursing

## Data Availability

The data used to support the findings of this study are included within the article.
